# Supplementary material for: Prevalence and factors associated with anemia among women of reproductive age in seven South and Southeast Asian countries: Evidence from nationally representative surveys
Source: PLoS One. 2020 Aug 13;15(8):e0236449. doi: 10.1371/journal.pone.0236449 (PMC7425935; doi:10.1371/journal.pone.0236449)
Supplement: S1 Table — (DOCX) [file pone.0236449.s001.docx]

**S1 Table. Data sources and sample size**

| **SN** | **Country** | **Source** | **Year** | **Total interviewed household (n)** | **Final sample size for WRA (n)** |
| --- | --- | --- | --- | --- | --- |
| 1 | Cambodia | DHS | 2014 | 15,825 | 11,286 |
| 2 | India | NFHS | 2016 | 601,509 | 679,445 |
| 3 | Maldives | DHS | 2016 | 6,050 | 6,653 |
| 4 | Myanmar | DHS | 2015 | 12,500 | 12,489 |
| 5 | Nepal | DHS | 2016 | 11,040 | 6,414 |
| 6 | Timor-Leste | DHS | 2016 | 11,502 | 4,201 |
| 7 | Bangladesh | DHS | 2011 | 17,141 | 5,676 |
