# Supplementary material for: Prevalence and factors associated with anemia among women of reproductive age in seven South and Southeast Asian countries: Evidence from nationally representative surveys
Source: PLoS One. 2020 Aug 13;15(8):e0236449. doi: 10.1371/journal.pone.0236449 (PMC7425935; doi:10.1371/journal.pone.0236449)
Supplement: Attachment — Submitted filename: Response to Reviewers.docx [file pone.0236449.s002.docx]

Manuscript ID: PONE-D-20-10365
Manuscript title: **Prevalence and factors associated with anemia among women of reproductive age in seven South and Southeast Asian Countries: evidence from nationally representative surveys**

Dear Editor,

We would like to thank the reviewers for the specific and needful comments. We have modified the paper in response to the extensive and insightful reviewers' comments. Sincerest thanks for providing us an opportunity to submit a revised copy of our manuscript entitled **"Prevalence and factors associated with anemia among women of reproductive age in seven South and Southeast Asian Countries: evidence from nationally representative surveys"** to esteemed Journal PLOS ONE. We appreciate the time and efforts that you and the reviewers have dedicated to providing your valuable feedback on our manuscript. We are grateful to the reviewers for their insightful comments on our papers. We believe the revisions have substantially improved our manuscript.

We have highlighted the changes within the manuscript where necessary. We hope that the manuscript is now suitable for publication. We look forward to hearing a positive response from you.

Here is a point-by-point response to the academic editor and reviewers' comments and concerns.

**Academic editor comments and concern**

1. Please ensure that your manuscript meets PLOS ONE's style requirements, including those for file naming. The PLOS ONE style templates can be found at

Response: Thank you for your suggestions. We have revised the formatting of our manuscript as per the PLOS ONE's manuscript body formatting guidelines.

2. We note that you have indicated that data from this study are available upon request. PLOS only allows data to be available upon request if there are legal or ethical restrictions on sharing data publicly. For information on unacceptable data access restrictions, please see <http://journals.plos.org/plosone/s/data-availability#loc-unacceptable-data-access-restrictions>.

In your revised cover letter, please address the following prompts:

a) If there are ethical or legal restrictions on sharing a de-identified data set, please explain them in detail (e.g., data contain potentially identifying or sensitive patient information) and who has imposed them (e.g., an ethics committee). Please also provide contact information for a data access committee, ethics committee, or other institutional body to which data requests may be sent.

b) If there are no restrictions, please upload the minimal anonymized data set necessary to replicate your study findings as either Supporting Information files or to a stable, public repository and provide us with the relevant URLs, DOIs, or accession numbers. Please see <http://www.bmj.com/content/340/bmj.c181.long> for guidelines on how to de-identify and prepare clinical data for publication. For a list of acceptable repositories, please see <http://journals.plos.org/plosone/s/data-availability#loc-recommended-repositories>.

We will update your Data Availability statement on your behalf to reflect the information you provide.

Response: Thank you for your valuable suggestions and comments. We used data of Demographic and Health Surveys that are publicly available and can be freely downloaded upon the formal request from the DHS website (<https://www.dhsprogram.com/data/available-datasets.cfm>). As per the guidelines and regulations of DHS, the dataset obtained by the authors for the purpose of statistical reporting and analysis specifically for this paper is not allowed to share in the public domain. However, we have uploaded the modified datasets in the online manuscript submission system to be only shared with the journal editor/reviewers for the purpose of the manuscript review process.

3. We note that Figure 1 in your submission contain map images which may be copyrighted. All PLOS content is published under the Creative Commons Attribution License (CC BY 4.0), which means that the manuscript, images, and Supporting Information files will be freely available online, and any third party is permitted to access, download, copy, distribute, and use these materials in any way, even commercially, with proper attribution. For these reasons, we cannot publish previously copyrighted maps or satellite images created using proprietary data, such as Google software (Google Maps, Street View, and Earth). For more information, see our copyright guidelines: <http://journals.plos.org/plosone/s/licenses-and-copyright>.

We require you to either (a) present written permission from the copyright holder to publish these figures specifically under the CC BY 4.0 license, or (b) remove the figures from your submission:

Response: Thank you very much for pointing out the important aspects of our manuscript. We assure you that the provided Fig1 contains a study area Map with anemia prevalence is copyright free. We created this map using Arc GIS software version 10.8 and base file of the administrative national and subnational bounderies for seven selected South and Southeast Asian (SSEA) country were obtained from the freely available copyright free resources Natural Earth (<http://www.naturalearthdata.com/>). The map was displayed by joining the prevalence of anemia with corresponding country. Also, we have mentioned this point within the manuscript, methods section of page number 9, line 187-189 within the revised manuscript.

Reviewers' comments:

Reviewer #1

Comment: This study aimed to assess the prevalence and factors associated with anemia among women of reproductive age (WRA) in seven selected South and Southeast Asian countries. This study was a secondary analysis of the seven selected South and Southeast Asian countries. During the 2012 World Health Assembly, the World Health Organization endorsed a target of a 50% reduction of anemia among women of reproductive age by 2025. Therefore, the study deals with a well-updated subject. Very few studies have utilized nationally representative data to investigate the prevalence and determinants of anemia among reproductive-aged women in the South and Southeast Asian context. With this, it adds important data.
Here are suggestions for the article.

Response: Thank you very much for your time spent and efforts to provide valuable comments on our manuscript.

Comments: Title Suggestion: "Prevalence and factors associated with anemia among women of reproductive age in seven South and Southeast Asian Countries: evidence from nationally representatives survey".
The insertion of the term "women of reproductive age" better specifies the study population. And, it would not lead to exceed the number of characters allowed.

Response: Thank you very much for your valuable suggestion. We have added the term "women of reproductive age" accordingly in the title and where necessary within the revised manuscript.

Abstract
Comments: In lines 24, 28 and 31 of the Abstract section there is an abbreviation, which is not prohibited, but should be avoided in this part of the text, according to the rules of the journal. The inclusion of the term "women of reproductive age" in its entirety would not exceed the number of characters in the section (300). Or, this can be a term added in the title, also without exceeding the character limit of the title which would better define the population studied.

Response: Thank you for pointing this out. We have replaced the WRA with "women of reproductive age" in the abstract section and where necessary in our revised manuscript.

Comments: The keywords "associated factors" and "South Southeast Asia" were not found in the MESH descriptors.
The text is brief, presents the objective of the study, includes substantial results, focuses on positive and non-negative findings.

Response: Thank you for your suggestion. According to the PLOS ONE's Journal manuscript body formatting guidelines, guidelines for keywords are not mentioned (<https://journals.plos.org/plosone/s/file?id=wjVg/PLOSOne_formatting_sample_main_body.pdf>). Also, most of the published articles have not used keywords in their article. Thus, in order to adhere to the manuscript body formatting guidelines, we removed all the keywords from the manuscript. However, we have submitted the appropriate keywords in the online manuscript submission form as per the PLOS one guideline.

Introduction
comments: The authors start from the macro idea, contextualize, and even identify the problem. The section is finished with the justification and the main objective of the work.

Response: Thank you very much.

Methodology
Comments: Experiments, statistics, and other analyses were performed to a high technical standard and were described in sufficient detail.

Response: Thank you.

Comments: Line 141 – change the unit gm/dl by g/dL for both hemoglobin <12g/dL and 11g/dL. Line 142 – change Homocue for Hemocue.

Response: Thank you for your comments. We have replaced the unit gm/dl with "g/dL" where necessary within the manuscript. Also, we have replaced the word Homocue with "Hemocue" in line 144 within the revised manuscript.

Comments: In lines 146, 147 and 148, the authors say that the predictor variables were chosen according to the literature review. However, in the introduction they say that several approaches at the population level have been taken over the years, such as micronutrient supplementation among adolescent girls and women, food fortification, nutritional education, counseling and orientation of an iron-rich dietary plan for at-risk populations. However, these are not variables evaluated in the study.
Response: Thank you. We agree with these constructive comments. We intended to explore the prevalence and factors associated with anemia among women of reproductive age in seven selected South and Southeast Asian countries. Based on the previous literature review regarding the risk of developing anemia among women in developing countries and the availability of data in the DHS dataset, we included household, environmental and individual factors as predictors' of anemia in our data analysis.. Meanwhile, we just underline the existing prevention and control strategy of anemia among women of reproductive age in the introduction section of our manuscript. These variables were not included because DHS does not collect this data. Even though these factors were not assessed in our study we believe highlighting these different approaches would provide the significance of the topic to the readers.

Comments: In lines 154 and 155 the authors need to explain better that the "wealth index" is a measure calculated by DHS itself and that it is based on the possession of consumer goods. It was not clear how it was obtained and, above all, how the categories were categorized. The understanding that it is a variable calculated by DHS is only possible if the reader searches the DHS Program website further.

Response: Thank you for pointing this out. We have added the *"wealth index calculation through principal component analysis (PCA) which is a standardized measure from household assets"* [1]. "*The wealth index variables were already included in the DHS dataset as five quintiles ranked as poorest, poorer, middle, richer, and richest. In this study, we further re-categorized as poor (poorest and poorer), middle and rich (richer and richest) for the analysis purpose"* [2] in page 7 and 8, line 156-161 within the revised manuscript.

Comments: Line 159 defines abbreviations upon first appearance in the text of ANC.
The variable iron intake is mentioned in lines 158 and 162, but do not explain what type of iron intake was this: supplementation acquired by women, given by some public policy, homemade fortification, dietetics? In the discussion (line 337), the limitation of not having evaluated food consumption data is mentioned. This variable is external intake? It is not clear.

We agree with these constructive comments. We have added the full form of ANC with "Antenatal checkup" in the manuscript in line 166 within the revised manuscript .

Yes, we have included iron supplementation during the most recent pregnancy in our analysis and revised the sentence accordingly in lines 170-171 within the revised manuscript. Indeed, Iron deficiency anemia is considered as one of the most common nutritional deficiency anemia among pregnant women. Iron folic acid supplementation program has been implemented in most of the south and Southeast Asian countries. Considering the significant role of *iron-folic acid supplementation* to prevent the anemia among women of reproductive age, we incorporated the iron intake variable in the analysis.

Comments: The research meets all applicable standards for the ethics of experimentation and research integrity.

Response: Thank you very much.

Results
comments: Table 2 -age group years - specify where women aged 35 entered. If it is in the third category express as ≥35 years.

Response: Thank you for pointing this out. We have corrected the age group years in Table 2 with "≥35 years".

Discussion
comments: In line 297, a study that demonstrates the prevalence of anemia in an age group quite different from that of the study (children).

Response: We removed the word children.
Comments: In lines 314 to 319, the authors return to the results of anemia prevalence in rural and urban areas, present a convergent reference to the finding, but do not discuss the possible causes of this.

Response: We agree with your valuable comments. We have added the relevant discussion in our revised manuscript in line 335-340 as "These convergent findings could be due to the selection of apparently healthy women living in rural areas and non-endemic areas for malaria and hookworm infestation prone areas in both countries. Moreover, in the Maldives the traditional staple foods consumed in rural households are more diversified and rich in iron contains compared to other countries in the same region. Such diversified food patterns in the rural areas of Maldives might have a positive role in meeting the adequacy of iron requirements for the reproductive age of women"[3,4].

Comments: In line 337, when they mention the absence of food intake data, they speak only of the intake of "mothers and children". They do not talk about the intake of non-pregnant women and the children's food intake would not reflect the mothers' food intake, because the eating pattern of such opposite age groups is different.

Response: Thank you for your comments. We have revised the sentence with "*this study could not consider the assessment of dietary factors among women of reproductive age that might have attributed to the risk of development of anemia"* in line 358-359 within the revised manuscript.

Conclusion
comments: The conclusion section responds to the proposed objectives and closes the subject by resuming the justification of the study and makes a brief prospective observation of what should be done from the findings.

Response: Thank you very much.

References
comments: Almost all references are from the last 10 years.
Review formatting of article references by placing them in Vancouver style. Examples of changes that need to be made include:
- The issues of journals should come in parentheses - examples - References 12, 15, 18, 19, 20.
- You do not put pp. before referring to the pages of journals.
- Reference 20 - quote in the supplement standards.
- References 14 and 25 do not contain the names of journals.
- On the pages of the references use only the unit or dozen on the final pages mentioned.

Response: We thank the reviewer for these important suggestions. We have corrected the references accordingly in our revised manuscript where necessary.

Reviewer #2:

Comments: Prevalence and factors associated with anemia among women in seven South and Southeast Asian Countries: evidence from nationally representatives survey
The study aimed to assess the prevalence and factors associated with anemia in women of reproductive age in seven selected countries in South and Southeast Asia. Based on the most recent data obtained from the Demographic and Health Surveys (DHS) of these seven countries, they analyzed data from 726,164 women. Multivariable binary logistic regression was performed to assess the factors associated with anemia among women in each country separately. Therefore, it is of relevance to public health study, which used nationally representative sample and appropriate statistical analysis. With minor adjustments it will be in conditions of publication.

Response: Thank you very much for your time spent and efforts to provide valuable comments on our manuscript.

Comments: The introduction presents prolix writing and with redundant information or that should be in another section. For example, right in the second line of the introduction it defines the cutoff points for anemia, which is suitable for is in methods (as it is also). There is an exaggerated exploration of the factors associated with anemia, as well as prevalence values in different situations. Much of this information would be better used in the discussion, situating the results found.

Response: Thank you for your suggestion. We have revised the sentences accordingly in the introduction section of our revised manuscript. The cut-off points for anemia have been removed from the introduction section in line 48-49 and moved to the methods section accordingly in line 143-144 within the revised manuscript.

Also, we added the information in discussion section as *" Stevens et al., (2013) reported the prevalence of anemia among non-pregnant women in South Asian countries declined slightly from an estimated 53% in 1995 to an estimated 47% in 2011, whilst the prevalence of anemia among pregnant women in the same region was almost stagnant (53% in 1995 and 52% in 2011)*" [5] in line 303-306 within the revised manuscript.

Comments: Line 159 – “…ever terminated pregnancy, ANC during pregnancy”: ANC must be defined a priori.

Response: We have revised and elaborate the sentence ever terminated pregnancy as *"Whether the respondent ever had a pregnancy that terminated in a miscarriage, abortion, and stillbirth"* in line 168-170 within the revised manuscript with track changes. Also, Antenatal checkup (ANC) during pregnancy refers to the *"number of antenatal visits during pregnancy"* in line 165-167 within the revised manuscript.

Comments: Line 160-161 – delete the second BMI: BMI was categorized into underweight BMI...

Response: Thank you for pointing this out. We deleted the second BMI.

Comments: Line 167 – “…analyzed using STATA/MP version 14.1” / Stata/MP, version 14.1

Response: We replaced the word STATA/MP with *"Stata/MP version 14.1"* accordingly in line 176.
Comments: Line 190 – It refers to figure 1, but the figure shown is named as figure 2. By the way, the title of this figure should be more intelligible (self-sufficient), so that the reader can understand what it is about without having to resort to the text.

Response: Thank you for your comments. We have revised the title Fig 1 with *"Prevalence of anemia among women of reproductive age by the geographical locations in seven South and Southeast Asia countries”* in line 200-201, which implied to display the geographical pattern and distribution of anemia prevalence rate using the map in the selected seven South and Southeast Asian country. Also, the map implies the study area being chosen for the analysis. Also, we have modified the Fig 2 with "*Prevalence of any form of anemia among women of reproductive age by country"* in line 235 within the revised manuscript.

Comments: Line 219 – “...women who didn't consume iron in all countries.” Use more precise wording: although insufficient, some amount they consumed.

Response: We have added the sentence in the line 230 within the revised manuscript with "*iron supplementation during most recent pregnancy"* to make more clear to the readers.

Comments: Line 227 – In the table title, write WRA in full.

Response: Thank you for pointing out this. We have included the full form WRA with *"women of reproductive age"* in the revised title of both Table 1 and Table 2.

Comments: Lines 247-248 – “The association of anemia among 15-49 years old women with environmental and individual factors was examined using the binary logistic regression model.” This has already been reported in methods.

Response: Thank you for your suggestion. We have deleted this sentence accordingly.

Comments: Line 249 - Women's having... / women's having...
Response: We have changed the word “Women's” with *"women's"* accordingly in the line 261.

Comments: Lines 265-266 – “age groups 15-24 years was positively associated with an increased likelihood of anemia compared to age groups of 25-34…” the term positively seems misused. If the variables are categorical (and not ordinal or continuous) and the highest prevalence was in the youngest age group, how was the relationship positive? He's confused. Confirm the adequacy of this term in other parts of the article.

Response: We thank the reviewer for constructive comments. We have corrected the sentence in the line 277-279 within the revised manuscript with "*the younger women (15-24 years) were more likely to be anemic compared with women in the age group of 25-34 years*"[6].

Comments: Line 296-301 – “The cross-sectional study from the DHS done in 27 Sub-Saharan Africa (SSA) between 2008 and 2014 found that the prevalence of anemia among children was 59.9% [33], which is slightly higher than our findings.” It makes no sense to compare data from children with those from women. If you want to use the information, make some bridge to make sense.
Response: Thank you very much for your insightful comments. We have removed these sentences and added the more relevant evidence from the previous findings in the line 310-313 with *“High prevalence of anemia among women from this region is attributed to the social and biological vulnerability within both household and community [7]. In SSEA countries, along with the nutritional anemia poverty and gender inequality also play a significant role in contributing anemia* [8]*”* within the revised manuscript.

Comments: Line 333 – “This study comprises of some limitations. First, due to the cross-sectional nature of data, this study…” Delete the last this study.

Response: Thank you. We have deleted the “last this study" and revised the sentence to make it clearer in line 354-355 within the revised manuscript with track changes.

Comments: Line 335 – “Second, Comparable data. / Second, comparable data…
Response: We revised the word accordingly in line 356.

Line 337 – “Third, dietary intake of mothers and children were not assessed…” the study does not address children.

Response: Thank you very for your constructive comments and feedback. We have revised the sentence with *"this study could not consider the assessment of dietary factors among women of reproductive age that might have attributed to the risk of development of anemia"* in line 358-359 within the revised manuscript. We removed the word "children" in line 363.

We look forward to hearing a positive response from you soon.

Thank you.

Sincerely

On behalf of all co-authors

Mr. Dev Ram Sunuwar, Dietician/Researcher

Armed Police Force Hospital, Kathmandu, Nepal

Email: [devramsunuwar@gmail.com](mailto:devramsunuwar@gmail.com)

**References**

1.	Rutstein SO, Johnson K. The DHS wealth index. Calverton, Maryland, USA: ORC Macro,. 2004.

2.	Gautam S, Min H, Kim H, Id HJ. Determining factors for the prevalence of anemia in women of reproductive age in Nepal : Evidence from recent national survey data. 2019; 1–17.

3.	Laxmi Pandey V, Mahendra Dev S, Jayachandran U. Impact of agricultural interventions on the nutritional status in South Asia: A review. Food Policy. 2016;62: 28–40. doi:10.1016/j.foodpol.2016.05.002

4.	Nath P, P.B. Gaddagimath. Horticulture and Livelihood Security - Google Books. [cited 4 Jun 2020]. Available: https://books.google.com.np/books?id=pEJLDwAAQBAJ&pg=PA127&lpg=PA127&dq=iron+rich+food+in+rural+area+of+maldives&source=bl&ots=MTYgRIUWyO&sig=ACfU3U2ouD86JvsAP8ZNcgCb1NwAW8cJZQ&hl=en&sa=X&ved=2ahUKEwjz7oOmqeXpAhUCbisKHTXfAaUQ6AEwAXoECAoQAQ#v=onepage&q=iro

5.	Stevens GA, Finucane MM, De-Regil LM, Paciorek CJ, Flaxman SR, Branca F, et al. Global, regional, and national trends in haemoglobin concentration and prevalence of total and severe anaemia in children and pregnant and non-pregnant women for 1995-2011: A systematic analysis of population-representative data. Lancet Glob Heal. 2013;1: e16. doi:10.1016/S2214-109X(13)70001-9

6.	Kibret KT, Chojenta C, D’Arcy E, Loxton D. Spatial distribution and determinant factors of anaemia among women of reproductive age in Ethiopia: A multilevel and spatial analysis. BMJ Open. 2019;9: 1–14. doi:10.1136/bmjopen-2018-027276

7.	Yang F, Liu X, Zha P. Trends in Socioeconomic Inequalities and Prevalence of Anemia Among Children and Nonpregnant Women in Low- and Middle-Income Countries. JAMA Netw open. 2018;1: e182899. doi:10.1001/jamanetworkopen.2018.2899

8.	Kassebaum NJ, Jasrasaria R, Naghavi M, Wulf SK, Johns N, Lozano R, et al. A systematic analysis of global anemia burden from 1990 to 2010. Blood. 2014;123: 615–24. doi:10.1182/blood-2013-06-508325
