# Supplementary material for: Prevalence and factors associated with anemia among women of reproductive age in seven South and Southeast Asian countries: Evidence from nationally representative surveys
Source: PLoS One. 2020 Aug 13;15(8):e0236449. doi: 10.1371/journal.pone.0236449 (PMC7425935; doi:10.1371/journal.pone.0236449)
Supplement: Attachment — Submitted filename: Response to Reviewers.docx [file pone.0236449.s003.docx]

Manuscript ID: PONE-D-20-10365R1
**Prevalence and factors associated with anemia among women of reproductive age in seven South and Southeast Asian Countries: evidence from nationally representative surveys**

Dear Editor,

Thank you very much for giving us an opportunity to submit a revised version of the manuscript entitled **"Prevalence and factors associated with anemia among women of reproductive age in seven South and Southeast Asian Countries: evidence from nationally representative surveys"** to reputed Journal PLOS ONE. We have revised the paper in response to the extensive and insightful reviewers' comments. We appreciate the time and efforts that you and the reviewers have dedicated to providing your constructive feedback on our manuscript. We believe the revisions have substantially improved our manuscript.

We have highlighted the changes within the manuscript where necessary. We hope that the manuscript is now suitable for publication. We look forward to hearing a positive response from you.

Here is a point-by-point response to the academic editor and reviewers' comments and concerns.

Reviewers' comments:

Reviewer #1

Comment: I believe that almost all comments were answered properly, which made the article clearer. Therefore, it can be published.
Response: Thank you very much for your constructive comments and suggestions.

Comments: I suggest that in reference number 14 the name of the journal BMC Women’s Health be included, as this suggestion has not been heeded. However, after this correction, there is no need to return the article to the reviewers for further evaluation.
Response: Thank you very much for your valuable suggestion. We have included the name of the journal *"BMC Women's Health"* in the reference number 14 in the revised version of the manuscript.

Reviewer #2:

Comments: Line 28-30: (Bangladesh DHS 2011, Cambodia DHS 2014, India NFHS 2016, Maldives DHS 2016, Myanmar DHS 2015, Nepal DHS 2016, Timor-Leste 2015)...
Change to: (Bangladesh, Cambodia, India, Maldives, Myanmar, Nepal and Timor-Leste)...
Response: We thank the reviewer. We have revised the sentence accordingly in the revised version of the manuscript, line 28-29.

Comments: Line 33: The combined prevalence of anemia among women of reproductive age in the seven selected South and Southeast Asian countries was…
Change to: The combined prevalence of anemia was…
Response: Thank you for your suggestion. We have revised the sentence accordingly with *"The combined prevalence of anemia was 52.5%, ranged from 22.7% in Timor-Leste to 63.% in the Maldives".* in revised manuscript line 32-33.

Comments: Line 66: …conducted by Balarajan Y., et al (2011) reported that […] urban or rural settings [6].
Change to: …conducted by Balarajan et al. [6] reported that [...] urban or rural settings.
Response: Thank you for pointing this out. We have revised the sentence with " *A pooled analysis conducted by Balarajan et al* [1] *reported that the risk of anemia among women living in the lowest wealth quintile, with no education, and also differed by urban or rural settings".* in revised manuscript line 65-66

Comments Line 113: Therefore, this study aims to identify […]: Start in a new paragraph.
Response: We have made a new paragraph of the sentence *"Therefore, this study aims to identify the prevalence and factors associated with anemia among WRA in seven selected SSEA countries"* in the revised manuscript line 112-113.

Comments: Line 143: […] anemia was defined […]: Put a comma between g/dL and respectively: (g/dL, respectively)
Response: Thank you for your comments. We have put a comma between g/dL and respectively with *"According to the WHO, for non-pregnant and pregnant women aged 15-49 years, any form of anemia was defined as hemoglobin concentration <12.0 g/dL, and 11 g/dL respectively"* in the revised manuscript line 141.

Comments: Line 144: Hemoglobin level was assessed using capillary blood and the HemoCue rapid testing technique […]
Wouldn't that be better? Hemoglobin level in capillary blood was assessed using the HemoCue rapid testing technique […]
Response: Thank you for your valuable suggestions. We have revised the sentences with "*Hemoglobin level in capillary blood was assessed using HemoCue rapid testing technique in all seven South and Southeast Asian countries. For further analysis of the outcome variable, the categories of anemia were further dichotomized as anemic and not anemic".* in the revised manuscript line 142-143.

Comments: Line 173: […] than one year. ANC visits during […]. In Portuguese, does not start sentence with abbreviations or numbers. I am not sure if there is this rule in English (I am not an English speaker).

Response: Thank you for pointing out this. We agree with you. We have replaced “ANC” with *"antenatal checkup"* in the revised manuscript line 170.

Comments: Line 206: Fig 1. Prevalence of anemia […]: Place after the next paragraph, in which fig. 1 is referred to for the first time.
Response: Thank you for your suggestion. We have moved the Fig 1 legend after line 211 in the consequent paragraph and text reference of Fig 1 has been also provided in the line 205.

Comments: Line 329: […]. Yang F et al. (2018) also reported […] middle-income countries [38].
Change to: […]. Yang et al. [38] also reported […] middle-income countries.
Response: We have changed the sentence with “*Yang et al.* [2] *also reported that the prevalence of anemia was observed higher among those individuals with low socioeconomic status from low and middle-income countries."* accordingly in the revised manuscript, line 317.

Comments: Other similar cases exist throughout the text and should be corrected.

Response: We thank the reviewer for constructive comments. We have corrected it wherever necessary throughout the revised manuscript.

We look forward to hearing a positive response from you soon.

Thank you.

Sincerely

On behalf of all co-authors

Mr. Dev Ram Sunuwar, Dietician/Researcher

Armed Police Force Hospital, Kathmandu, Nepal

Email: [devramsunuwar@gmail.com](mailto:devramsunuwar@gmail.com)

References

1.	Balarajan Y, Ramakrishnan U, Özaltin E, Shankar AH, Subramanian S V. Anaemia in low-income and middle-income countries. Lancet. 2011;378: 2123–2135. doi:10.1016/S0140-6736(10)62304-5

2.	Yang F, Liu X, Zha P. Trends in Socioeconomic Inequalities and Prevalence of Anemia Among Children and Nonpregnant Women in Low- and Middle-Income Countries. JAMA Netw open. 2018;1: e182899. doi:10.1001/jamanetworkopen.2018.2899
