# Supplementary material for: Prevalence and factors associated with anemia among women of reproductive age in seven South and Southeast Asian countries: Evidence from nationally representative surveys
Source: PLoS One. 2020 Aug 13;15(8):e0236449. doi: 10.1371/journal.pone.0236449 (PMC7425935; doi:10.1371/journal.pone.0236449)
Supplement: Attachment — Submitted filename: Response to Reviewers.docx [file pone.0236449.s004.docx]

Manuscript ID: PONE-D-20-10365R2
**Prevalence and factors associated with anemia among women of reproductive age in seven South and Southeast Asian Countries: evidence from nationally representative surveys**

Dear Editor,

We would like to thank you for allowing us to submit a revised copy of our manuscript entitled **"Prevalence and factors associated with anemia among women of reproductive age in seven South and Southeast Asian Countries: evidence from nationally representative surveys"** to reputed Journal PLOS ONE. We appreciate the time and effort that you and the reviewers have dedicated to providing your valuable feedback on our manuscript. We are grateful to the reviewers for their insightful comments on our papers. We believe the revisions have substantially improved our manuscript.

We have replaced the word "multivariable regression model" with "multiple logistic regression model" in the text and Tables when referring to multiple regression analyses. We hope that the manuscript is now suitable for publication.

We look forward to hearing a positive response from you.

Thank you.

Sincerely

On behalf of all co-authors

Mr. Dev Ram Sunuwar, Dietician/Researcher

Armed Police Force Hospital, Kathmandu, Nepal

Email: [devramsunuwar@gmail.com](mailto:devramsunuwar@gmail.com)

Here is a response to the academic editor's comments and concerns.

Comment: The authors have made all changes requested by the reviewers. However, I have noted the use of the word "multivariable" instead of "multiple" regression models. Thus, please replace "multivariable" by "multiple" in the text and Tables when referring to multiple regression analyses.
Response: Thank you very much for your constructive comments and suggestions. We have replaced the word "multivariable regression model" with "*multiple logistic regression model"* in the text and Tables when referring to multiple regression analyses within the revised version of the manuscript.
